# Validation of Strategic Memory Advanced Reasoning Training as an efficient and effective approach to treating warfighters with persistent cognitive complaints associated with mild traumatic brain injury

**DOI:** 10.3389/fneur.2025.1541894

**Published:** 2025-04-09

**Authors:** Andrew J. Darr, Angela Basham, Jessica L. Ryan, Melissa Caswell, Juan Lopez, Jennifer Zientz, Erin Venza, Ida Babakhanyan, Sandra Chapman, Jason M. Bailie

**Affiliations:** ^1^Traumatic Brain Injury Center of Excellence (TBICoE), Silver Spring, MD, United States; ^2^General Dynamics Information Technology, Silver Spring, MD, United States; ^3^Intrepid Spirit, Naval Hospital Camp Pendleton, Oceanside, CA, United States; ^4^Tampa VA Research and Education Foundation at James A. Haley Veterans’ Hospital, Department of Emergency Medicine, University of South Florida, Tampa, FL, United States; ^5^Center for BrainHealth, Behavioral and Brain Sciences, The University of Texas at Dallas, Dallas, TX, United States

**Keywords:** mild traumatic brain injury, concussion, post concussive symptoms, cognitive rehabilitation, military health

## Abstract

**Introduction:**

This study directly compared the relative effectiveness of Strategic Memory Advanced Reasoning Training (SMART), which focuses on metacognitive strategies, to a traditional cognitive rehabilitation (CR) program previously developed and validated for the Study of Cognitive Rehabilitation Effectiveness study (SCORE), in treating warfighters with a history of mild traumatic brain injury (mTBI) and persistent post-concussive symptoms (PCS).

**Methods:**

A total of 148 active-duty service members (SMs) were recruited for this randomized controlled trial (RCT). Participants were randomly assigned to either the SMART (*n* = 80) or SCORE (*n* = 68) intervention arms. Outcome measures were administered at the start (T1) and end of treatment (T2), and at 3 months post-treatment (T3). Only participants with data from all timepoints and adequate performance validity (SMART: *n* = 51; SCORE: *n* = 43) were used in analyses. The primary outcome measure was the Global Deficit Scale (GDS), a composite of seven different objective measures of cognitive performance. Secondarily, participants completed the Neurobehavioral Symptom Inventory (NSI) and Key Behaviors Change Inventory (KBCI) self-report measures of post concussive symptoms (PCS). Lastly, a cost effectiveness analysis (CEA) was performed directly comparing the relative efficiencies of the two CR interventions.

**Results:**

Mixed Analysis of Variance (ANOVA) showed a significant decrease in GDS scores from T1 to T3 (*p* < 0.001, *η_p_*^2^ = 0.217), irrespective of intervention type (*p* = 0.986, *η_p_*^2^ = 0.000). The greatest improvement occurred between T1 (SMART: *M* = 0.70, SD = 0.79; SCORE: *M* = 0.70, SD = 0.72) and T2 (SMART: *M* = 0.29, SD = 0.58; SCORE: *M* = 0.29, SD = 0.40), with scores plateauing at T3 (SMART: *M* = 0.28, SD = 0.52; SCORE: *M* = 0.29, SD = 0.57). Similarly, there was a significant decrease in NSI scores over the same period (*p* < 0.001, *η_p_*^2^ = 0.138), regardless of intervention type (*p* = 0.412, *η_p_*^2^ = 0.010). Additionally, treatment improved patient perceived functionality (KBCI) from T1 to T2 and these gains remained stable at T3 (*p* < 0.001, *η_p_*^2^ = 0.377). CEA revealed SMART represented a 60% reduction in cost compared to SCORE.

**Discussion:**

This study demonstrates that SMART is an effective strategy for reducing cognitive deficits and PCS in SMs with a history of mTBI, producing comparable outcomes to a traditional CR program in less time and with improved cost efficiencies.

## Introduction

Traumatic brain injury (TBI) and warfighter brain health remains a major health concern for the United States Military Health System (1, 2). The Department of Defense (DoD) has recognized the critical importance of addressing these issues through programs like the Warfighter Brain Health Initiative, which aims to optimize brain health and improve outcomes following combat TBI among service members (SMs). This concern is underscored by the significant number of TBIs reported among warfighters, with nearly 505,896 cases documented between 2000 and early 2024, of which approximately 82% were classifiable as ‘mild’ (3). Mild traumatic brain injury (mTBI) is the most common type of brain injury, and is generally associated with good recovery; however, a percentage of individuals will have persistent symptoms, with cognitive complaints being among the most common (4). Individuals with chronic post-concussive symptoms (PCS), particularly in military and veteran populations, frequently report cognitive symptoms (5). Unfortunately, many SMs have a history of multiple head injuries as well as psychological co-morbidities, which can negatively influence recovery and increase the likelihood of long-term cognitive deficits (6, 7, 8). Moreover, PCS may limit a warfighter’s ability to complete their duties and, on a larger scale, limit overall force readiness.

Due to heightened awareness and the large number of SMs experiencing chronic PCS, there is increased importance placed on identifying or developing effective treatment options. An effective rehabilitation program to remediate cognitive deficits in injured SMs would have direct impact to force readiness and may decrease the long-term health utilization costs from both active duty SMs and veterans. When determining optimal treatment for SMs, it is important to recognize that a tenant of military medicine is to return injured warfighters to full-unrestricted duty status. In terms of mTBI, rehabilitation of cognitive deficits should be focused on the complex mental tasks necessary for high-pressure combat situations and other military operations. The expectation is that warfighters be cognitively ready, meaning they need to be mentally prepared to handle the demanding, unpredictable, and high-stress situations they may encounter in the field. Thus, cognitive readiness is crucial for mission success, as well as the overall health and safety of SMs. The recognized core components of cognitive readiness are aspects of higher-order cognitive reasoning: situational awareness, problem solving, metacognition, decision-making, adaptability, and creative thinking. Importantly, all military personnel must be able to translate the techniques and strategies learned in treatment to novel environments, including adapting to new technological capabilities, and making split-second potentially life-threating decisions, all of which are characteristic of both combat and training environments (9).

Another challenge is treatment generalizability from civilian and veteran communities to active duty SMs. This may pose some unique and unexplored factors, as the goal of treatment is not remediation of common activities of daily living (e.g., remembering appointments) but military readiness needed for worldwide deployability. Current cognitive rehabilitation (CR) strategies focus primarily on application of techniques originally developed for basic and instrumental activities of daily living that are impaired after more severe brain injury. CR has typically focused on compensating for subjective and functional cognitive complaints (10, 11). These treatments have shown relatively modest success in management of symptoms in SMs, who primarily have mild brain injuries and are requesting assistance with higher level cognitive functions (e.g., selective attention and complex decision-making) associated with military operations (12). The Study of Cognitive Rehabilitation Effectiveness (SCORE) randomized clinical trial (13) used various therapies in an active-duty population: (i) psychoeducation, (ii) independent self-administered computer-based CR, (iii) therapist-directed manualized CR, and (iv) therapist-directed CR integrated with cognitive behavioral therapy (CBT) psychotherapy. They found that treatment arms that included therapist-directed CR had superior outcomes to the other treatment arms as measured by self-reported day-to-day cognitive functioning. Though promising, the results of the SCORE trial suggest that traditional therapist-based CR interventions are far from optimal. In the SCORE therapist-based CR intervention, only 23.3% of the sample had a meaningful reduction in symptoms after extensive treatment. Another limitation of traditional therapist-directed CR treatment is duration of treatment. For example, the therapist-directed manualized CR used in the SCORE randomized clinical trial included 10 h a week of treatment for 6 weeks (13). A similar CR treatment (14) lasted 10 weeks in duration. Many published CR programs are very time-consuming which affects the time of return to duty and may hinder its ability to be executed in military medical settings with limited resources or in military populations who are frequently relocating geographical sites (e.g., Special Forces).

Strategic Memory Advanced Reasoning Training (SMART) represents a possible alternative to traditional CR, with the potential to overcome many of the limitations of traditional therapist-based CR when applied to treatment of mTBI in active duty SMs. SMART is an evidence-based, manualized cognitive training protocol focused on enhancing top-down executive functioning via three metacognitive strategies: strategic attention, integrated reasoning, and innovation (15). Numerous randomized clinical trials with various populations have demonstrated improved cognitive performance and emotional wellbeing using SMART strategies, which focus not on compensatory strategies for daily living but more advanced complex reasoning skills that are essential for warfighter readiness. SMART has shown efficacy in improving cognitive functioning in both neurologically injured individuals as well as healthy adults (16). In randomized control trials, SMART has been shown to enhance complex reasoning, working memory and innovative cognition in healthy adults (15, 17, 18). In these studies, compared to two different control conditions, SMART was linked to improved neural gains in both the fronto-parietal and cingulo-opercular brain networks, areas involved in speed based reasoning and essential for warfighter cognitive functioning in unpredictable combat situations. Multiple clinical trials have demonstrated that SMART improves mental agility, focus, psychological wellbeing, and functional life outcomes in individuals with mild cognitive deficits following TBI, as well as healthy individuals (15, 16, 19–21). Notably, SMART also has a shorter duration of treatment time than current CR treatments and may be administered by a wide array of rehabilitation therapists (e.g., speech therapists, occupational therapists, psychologists), which are already employed across the military health system. Perhaps most importantly to SMs, there is encouraging evidence that training with SMART may translate to gains on the battlefield. In a translation study involving military personnel, investigators showed significant improvement in cognitive functioning, evidenced by better performance on measures of integrated reasoning and innovation (22). Furthermore, this improved performance occurred following a shortened intervention period consisting of just 6–10 h over a 4-week period (22).

Several studies have demonstrated that SMART can influence some of the neurophysiology underpinning higher order cognitive functioning. A study using structural and resting-state functional MRI found that SMART increased cortical thickness in four right prefrontal regions and decreased resting-state connectivity in these areas in individuals with chronic TBI, compared to controls (19). Another study demonstrated that training with SMART induced changes in whole-brain neural networks, and that this neuroplasticity was associated with improved functioning (23). Using a graph-theoretical approach, investigators showed that SMART was associated with a reorganization of modular brain networks in individuals with a history of TBI. Specifically, they were able to show that SMART reduced modularity due to increased connectivity between modules, yielding increased global and local processing efficiencies (23). SMART has also been associated with increased blood flow to the brain. In a study of reasoning training in veterans and civilians with TBI and persistent (mild) functional deficits, investigators found an association between increased cerebral blood flow (CBF) and improved reasoning following SMART (24). This increased CBF was observed in the left inferior frontal region, the left insula, and the bilateral anterior cingulate cortex, areas associated with cognitive control performance and executive function (24). These same investigators also showed increased delivery of regional cerebral blood flow (rCBF) bilaterally in the precuneus (24), a region previously shown to have hypometabolism linked to increased severity of TBI (25). Based on the neurovascular coupling principle, the results of an increase in CBF following SMART in the bilateral precuneus are suggestive of improved neural health in this key region linked to psychological health. Notably, reduced neuronal activity in the precuneus has been reported in individuals with comorbid TBI and post-traumatic stress disorder (26). Lastly, SMART was shown to reduce depressive symptoms in TBI regardless of whether the training directly targeted specific psychiatric symptoms. Notably, investigators observed that decreased Beck Depressive Inventory scores within the depressive-symptoms group was associated with improvements in scores for PTSD, as well as TBI symptom awareness and functional status (27).

Given SMART’s effectiveness (as demonstrated by reduced cognitive deficits, improved performance on cognitive tasks, and enhanced physiological functioning), reduced time of treatment compared to traditional CR, and the potential to improve cognitive readiness for warfighters, a detailed exploration of SMART’s effectiveness in rehabilitating active duty SMs with mTBI is warranted. The primary objective of this study was to evaluate SMART treatment for rehabilitation of active duty SMs with chronic cognitive deficits following a mTBI. The key question was whether a cognitive-control, top-down training protocol can achieve better cognitive health, psychological health, and functional life outcomes than traditional cognitive rehabilitation. We hypothesized that SMART would result in a larger reduction in cognitive deficits as measured by the Global Deficit Scores (GDS) compared to a traditional (CR) program (SCORE). Secondarily, participants completed the Neurobehavioral Symptom Inventory (NSI) and Key Behaviors Change Inventory (KBCI) self-report measures of post concussive symptoms (PCS). Finally, a cost effectiveness analysis (CEA) comparing the relative costs and outcomes of SMART and SCORE was performed. This approach to evaluating rehabilitation programs can help guide decisions on resource allocation by identifying the most efficient program for achieving desired cognitive outcomes. We hypothesized that the differences in treatment duration and patient throughput of these independent cognitive rehabilitation programs would impact the overall cost of treatment.

## Materials and methods

### Design

This prospective randomized clinical trial (RCT) compared the effectiveness of two cognitive rehabilitation approaches—SMART and SCORE—among active-duty military personnel with persistent cognitive complaints following mTBI.

### Participants

A total of 148 active-duty service members (SMs) were recruited from a large military treatment facility. Participants had a history of at least one mTBI based on the DoD diagnostic criteria (28). Participants had to be greater than 3 months from injury and have ongoing chronic cognitive complaints defined as moderate or greater severity on one of the four cognitive symptoms from the Neurobehavioral Symptom Inventory (NSI). TBI diagnosis was confirmed with the Ohio State University Traumatic Brain Injury Identification Method (OSU TBI-ID) (29–31) as well as documentation in electronic medical records (e.g., AHLTA/MHS GENESIS).

**Figure 1:**
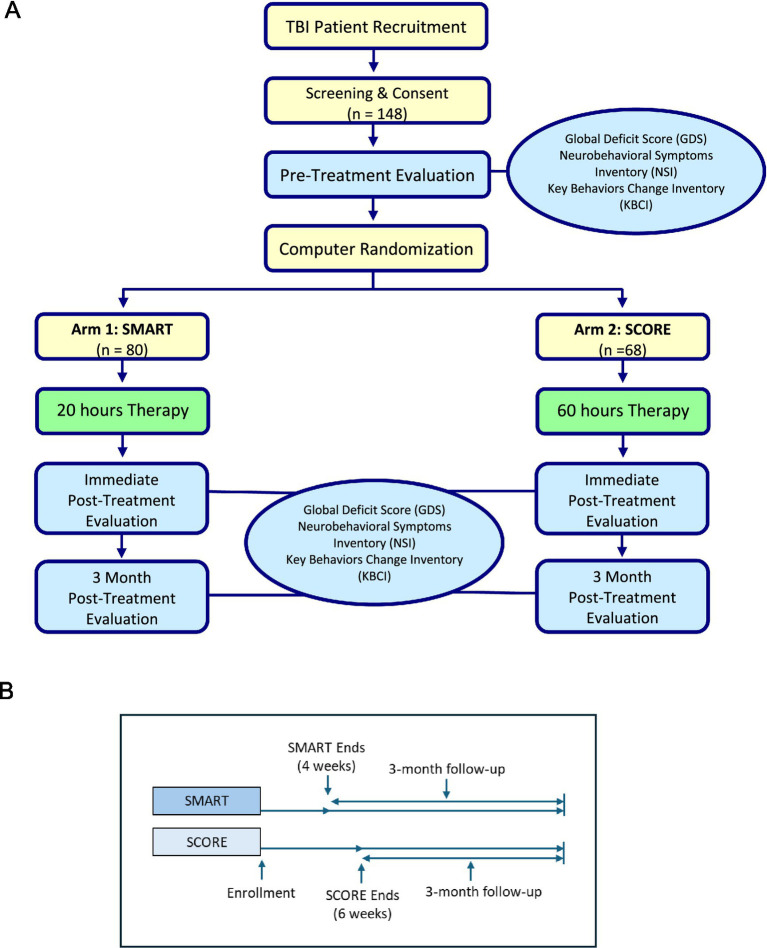
**(A)** Flow of participants through the study protocol. After screening and consent, a pre-treatment evaluation was completed to determine baseline functioning. For each treatment, evaluations were completed immediately post-treatment (at approximately 4 weeks from baseline for SMART and 6 weeks from baseline for SCORE) and at 3 months post-treatment. **(B)** Details the study procedure timeline.

Figure 1 shows the flow of participants through the study protocol. Participants were grouped into cohorts and each cohort was randomized into one of the two targeted interventions (SMART and SCORE). The SMART intervention was able to accommodate cohorts of 2–6 participants and the SCORE intervention was able to accommodate 2–4 participants. Ultimately, 80 participants were randomized to SMART and 68 participants were randomized to SCORE. After 4 weeks of the SMART treatment, 90% (*n* = 72) of participants completed the immediate post-treatment assessment (T2). After 6 weeks of the SCORE treatment, 82.4% (*n* = 56) of participants completed the immediate post-treatment assessment (T2). The final post-assessment (T3) was completed 3 months after the treatment intervention; 77.5% (*n* = 62) of SMART participants completed T3 while 75% (*n* = 51) of SCORE participants completed T3.

### Performance validity testing (PVT)

To ensure cognitive assessments were valid and suitable for interpretation, this study relied on PVT designed to detect atypical cognitive performance that is not indicative of known and/or legitimate cognitive impairment. This was done to ensure that patient performance on tasks was reflective of their true abilities and not attributable to other factors (e.g., malingering, lack of effort). PVT included the Rey-15 Item Test and Recognition Trial with a threshold of <26 (32), the Delis Kaplan Executive Function System (DKEFS) Color Naming and Word Reading combined score with a cut off of <6 (33), as well as DKEFS Visual Scanning, Number Sequencing, and Letter Sequencing tests with a combined score of <15 (34). Participants were excluded from the study if they failed two of the three tests at any of the assessment time points. A total of 19 participants were excluded based on failed PVT testing.

### Intervention arms

#### Strategic Memory Advanced Reasoning Training (SMART)

SMART is a proprietary, manualized cognitive training program focused on three core metacognitive strategies: Strategic Attention, Integrated Reasoning, and Innovation. Table 1 provides an overview of the SMART module topics, session structure, and example activities. Although the full session-by-session content is proprietary, this overview clarifies how we delivered SMART in a four-week timeframe (total of ~20 h). Week 1 focused on Strategy Education with five 1-h sessions introducing the core strategies. Weeks 2–4 focused on Strategy Application through a combination of group sessions and individual sessions that guided participants in applying the strategies to real-life tasks, with emphasis on military-relevant scenarios. Over the 4-week period, there were 14 h of group training and 6 h of individual training for a total of 20 h. All treatment sessions were completed by a licensed speech language pathologist (SLP). Each participant was provided a treatment manual with worksheets and educational activities. The SMART manual includes a guide to strategy application and goal setting prompts for individual and group activities and implementation assignments.

**Table 1:** SMART training delivery.

Timeframe	Frequency/format	Concepts	Examples
Days 1–5	60 min/day Group sessions	Strategy instruction 1. Strategic attention: Block irrelevant information and focus on key data. 2. Integrated reasoning: Identify big-picture concepts to guide real-life decisions. 3. Innovation: Generate multiple solutions/perspectives to strengthen mental flexibility.	Calibrate mental energy to accomplish top two tasks prioritized by amount of cognitive effort required (vs time required); single-tasking to optimize focus and clearly filter unnecessary data points; calm the brain and prime its energy via purposeful disconnection from technology and people. Abstract key concepts from complex information, combine with existing knowledge, and identify impact for meaningful application to guide decision-making and problem solving in real time. Proactively identify numerous options/solutions as practical courses of action; seeking perspectives different from one’s own; reframing mistakes and cultivating a culture tolerant of trial-and-error; challenging status quo to explore curiosity and execute tasks in novel ways.
Days 6–20	60 min/day 9 days group 6 days individual	Strategy application Group sessions: Practice strategies by applying to real-life scenarios in a cohesive way. Individual sessions: Use a BrainHealth Goal template to set, track, and work toward personal goals related to daily life and brain health.	Managing the massive volume of daily taskers: Proactively prioritizing tasks that require most cognitive effort (focus/concentration) by identifying the goal and impact of each; recognizing optimal ‘prime time’ and dedicating uninterrupted period to accomplish each task; taking brain breaks at transition points to calm and prime the brain with recharged mental energy; establishing a stopping point by which accomplishment has been achieved. Personal goal to improve family relationships: Proactively asking novel questions at the dinner table to engage in deeper level conversation; practicing perspective-taking when discussing difficult issues; limiting technology usage during family outings; planning new weekend adventures.

#### Study of Cognitive Rehabilitation Effectiveness (SCORE)

The traditional CR program was a clinician-directed, manualized intervention previously developed and validated for the SCORE trial (13). In this functionally oriented program, performance is improved through repetition, errorless learning, and gradually increasing task difficulty and complexity in a structured systematic approach. The intervention lasted 6 weeks, at 10 h per week, for a total of 60 h. Each week, the intervention included 5 one-hour individual sessions, two of which were focused on compensatory strategies and three of which were focused on restorative strategies. There were also two 1-h weekly group therapy sessions focused on compensatory strategies, and 3 h of weekly computer-based work with the Attention Process Training-3 program (APT-3; (35)). All computer-based sessions were proctored by clinic staff who recorded performance and provided positive reinforcement of participation and effort. A licensed SLP conducted treatment. Each participant received a treatment manual comprised of educational materials and integrated individual and group activities and assignments.

### Outcome measures

#### Global Deficit Score (GDS)

A GDS was calculated using methods adapted from previously published work (36, 37). GDS was calculated from 7 cognitive measures: Hopkins Verbal Learning Test-Revised Total Recall (HVLT-TR) and Delayed Recall (HVLT-DR) (38); Delis Kaplan Executive Functioning System Color Word Conditions 3 and 4 and Trail Making Conditions 4 (39); Paced Auditory Serial Addition Test (PASAT) (40, 41); and the Symbol Digit Modality Test (SDMT) (42). By aggregating deficits across multiple tests, the composite score (i.e., GDS) potentially enhances the detection of subtle cognitive deficits (particularly important in this population) that might not be apparent in individual assessments.

Demographically corrected t-scores (*M* = 50, SD = 10) for each cognitive measure were converted to deficit scores. T-score to deficit score conversions were as follows: >40 = 0, 35–39 = 1, 30–34 = 2, 25–29 = 3, 20–24 = 4, <20 = 5. The 7 deficit scores were averaged to calculate GDS. A score of 0 represents no impairment and higher scores represented greater cognitive impairment; based on prior studies, scores ≥0.5 signify impaired cognitive performance.

#### Neurobehavioral Symptom Inventory (NSI)

The NSI is a 22 item self-report questionnaire that assesses PCS (43). The questionnaire is a preferred measure by the DoD and Veterans Affairs. It is well-validated in research studies on TBI and PTSD in military populations, it has known reliability and validity, as well as guidelines for assessment of change (44–46). The NSI categorizes symptoms into four primary domains or clusters: physical, cognitive, affective, and sensory (46).

#### Key Behaviors Change Inventory (KBCI)

The KBCI is a self-reported measure of functional deficits that are associated with a TBI. It is a 64-item questionnaire that assesses behavioral areas such as lack of motivation, difficulties communicating, lack of insight into difficulties, and relationship problems (47). The scale measures eight domains of functioning (inattention, impulsivity, interpersonal, apathy, somatic, unawareness, communication, and emotional) and the total score which is an average of the scales; all scores represent a standardized t-score based on previously published normative data. The instrument has good content and construct validity and an internal consistency reliability of 0.82–0.91.

### Statistical analysis

The differences between treatment groups sample characteristics including demography, military information and co-variates were evaluated using Students ‘t’ and chi-square tests for continuous and categorical variables, respectively. The Mann–Whitney U test was used for between groups comparisons where continuous variables were not normally distributed. Mixed-effect analysis of variance (ANOVA) was conducted for the primary hypothesis with treatment modality (SMART vs. TCR) as the independent variable. This approach took into consideration between-subject variance based on randomized group, as well as within-subject variance for repeated measures. Primary analyses were based on intention to treat, and we leveraged statistical power of multilevel modeling to handle missing follow-up data. Outcomes were assessed as change in GDS over time. The same procedure was completed for the NSI total score and KBCI total score. A significant *p*-value of 0.05 was used to assess the main and interaction effects (partial eta squared). Where analyses consisted of timepoints 1 and 2 only, within-group related samples t tests were performed, and Cohen’s d effect sizes reported.

### Cost effectiveness analysis (CEA)

We completed a cost effectiveness analysis (CEA) comparing SMART and SCORE. CEA is a way to evaluate both the costs and health outcomes of one or more interventions by estimating how much it costs to gain a unit of a health outcome, in this case, improved cognitive and behavioral functioning. As outcomes were equivalent, the CEA focused on costs alone. The largest direct cost of the interventions was the time of the speech language pathologists (SLPs) and the warfighters who were treated while on duty. All other direct costs (e.g., equipment) were similar between the two treatments. Time costs for the SLP included individual and group therapy; for group treatment hours, the time was divided by number of participants in each group to find the mean SLP time spent in group therapy per participant. Warfighter time costs included individual and group therapy as well as homework and transportation. Mean total costs per participant were calculated as (mean hourly wage of a SLP multiplied by mean hours spent on that treatment per participant) plus (mean hourly wage of warfighter multiplied by mean hours spent on that treatment). The hourly mean wage of a SLP based in California where the study was executed was derived from the U.S. Bureau of Labor Statistics (BLS). The hourly mean wage of the warfighter was based on participant’s rank and the DoD’s monthly basic pay chart. All costs are reported in 2022 United States (U.S.) dollars.

## Results

### Demographics and TBI history

Demographic and injury history data are provided in Tables 2, 3, respectively. There were no statistical differences between the two treatment groups in terms of age, gender, race, ethnicity, education level, marital status, or current living situation (Table 2). For both groups, the participants were typically white/Caucasian, married men, in their mid-thirties (Table 2). In terms of TBI History, the treatment groups did not differ on any of the key variables from the Ohio State University TBI History; with the exception that the SMART condition had more years participating in activities related to repetitive head injuries (e.g., playing contact sports) (Table 3). Both samples had a median of 4 mTBIs in their lifetime with median time since last mTBI being more than 5 years (Table 3). Pre-treatment (T1) the groups did not differ in their neurobehavioral symptom severity (NSI), TBI related functional difficulties (KBCI), depression (Patient Health Questionnaire-8), sleep quality (Pittsburgh Sleep Quality Index-PSQI), or post-traumatic stress levels (Post-Traumatic Stress Disorder Checklist-PCL-M), nor did the groups differ in terms of their number of past combat deployments, combat exposure severity (Combat Exposure Scale-CES), lifetime blast exposure (Blast Exposure Threshold Study-BETS), or premorbid functioning (Test of Premorbid Functioning) (Table 3).

**Table 2:** Demographics and injury history by intervention.

Variable	SCORE (*n* = 43)	SMART (*n* = 51)	*p* val
Age (Mdn, Range)	35.0 (19–50)	36.0 (21–52)	0.273^$^
Sex (*n*, %)			0.829^#^
Male	40 (93.0%)	48 (94.1%)	
Female	3 (7.0%)	3 (5.9%)	
Race (*n*, %)			0.706^#^
White/Caucasian	35 (81.4%)	40 (78.4%)	
Black/African American	4 (9.3%)	3 (5.9%)	
American Indian/Alaska Native	1 (2.3%)	1 (2.0%)	
Asian	0 (0.0%)	2 (3.9%)	
Native Hawaiian/Other Pacific Islander	0 (0.0%)	1 (2.0%)	
Not reported	3 (7.0%)	4 (7.8%)	
Ethnicity (*n*, %)			0.220^#^
Hispanic/Latino	8 (18.6%)	11 (21.6%)	
Not Hispanic/Latino	34 (79.1%)	33 (64.7%)	
Unknown	0 (0.0%)	1 (2.0%)	
Not reported	1 (2.3%)	6 (11.8%)	
Education (*n*, %)			0.093^$^
HS diploma/GED	15 (34.9%)	12 (23.5%)	
Some college (1–3 yrs./technical school)	20 (46.5%)	22 (43.1%)	
College graduate (4 yrs. or more)	3 (7.0%)	8 (15.7%)	
Some graduate school	4 (9.3%)	4 (7.8%)	
Graduate/Professional program	1 (2.3%)	5 (9.8%)	
Marital status (*n*, %)			0.357^#^
Never married	11 (25.6%)	7 (13.7%)	
Married	28 (65.1%)	36 (70.6%)	
Separated	2 (4.7%)	2 (3.9%)	
Divorced	2 (4.7%)	6 (11.8%)	
Living situation (*n*, %)			0.278^#^
Live alone	6 (14.0%)	7 (13.7%)	
Live with friend(s) or roommate(s) or cohabitating	9 (20.9%)	10 (19.6%)	
Live with spouse and/or other family member(s)	25 (58.1%)	34 (66.7%)	
Other	3 (7.0%)	0 (0.0%)	
Number of deployments (Mdn, Range)	3.0 (0–13)	3.0 (0–25)	0.056^$^

$ Mann–Whitney U Test.

# Pearson Chi-Square Test.

**Table 3:** TBI factors and clinical factors by intervention pre-treatment.

Variable	SCORE (*n* = 43)	SMART (*n* = 51)	*p* val
OSU TBI-ID			
Total mTBI (Mdn, Range)	4 (1–11)	4 (1–13)	0.692^$^
LOC (Mdn, Range)	1 (0–7)	1 (0–5)	0.578^$^
Age first TBI (M, SD)	16.9 (7.0)	17.4 (7.7)	0.741^^^
Yrs last TBI (Mdn, Range)	5 (0–40)	7.5 (0–24)	0.435^$^
Per rep head inj (Mdn, Range)	2 (0–5)	2 (0–9)	0.149^$^
Total yrs. rep head inj (Mdn, Range)	8 (0–23)	12 (0–31)	0.020^$^
Blast TBI (Mdn, Range)	1 (0–6)	1 (0–7)	0.815^$^
PCL-M (Mdn, Range)	42.0 (17–85)	43.0 (19–77)	0.985^$^
PHQ-8 total (M, SD)	11.4 (5.8)	10.9 (4.7)	0.649^^^
NSI (M, SD)	39.5 (15.3)	37.2 (12.3)	0.425^^^
KBCI Total (M, SD)	70.2 (8.9)	69.0 (6.6)	0.456^^^
PSQI Total (M, SD)	12.52 (4.13)	11.88 (3.95)	0.448^^^
CES (Mdn, Range)	20.0 (7–32)	19.5 (7–31)	0.316^$^
BETS GBEV (Mdn, Range)	532,403 (1,171–469,658,227)	2,025,895 (3,172–246,153,118)	0.093^$^
TOPF (Mdn, Range)	102.0 (80–117)	106.5 (71–123)	0.126^$^

M, Mean; Mdn, Median; SD, Standard Deviation; *p*, *p* value (alpha <0.05); PCL-M, Posttraumatic Stress Disorder Checklist – Military version; PHQ-8, 8-item Patient Health Questionnaire; NSI, Neurobehavioral Symptom Inventory; KBCI, Key Behaviors Change Inventory; PSQI, Pittsburgh Sleep Quality Index; CES, Combat Exposure Scale; BETS GBEV, Blast Exposure Threshold Survey Generalized Blast Exposure Value; TOPF, Test of Premorbid Functioning.

$ Mann–Whitney U Test.

^ Independent Samples T Test.

The number of hours each participant spent with a clinician is detailed in Table 4. Consistent with the study design, the participants who completed SMART had significantly fewer hours in treatment than those in SCORE. For SCORE, the participants completed on average 26.6 of 30 h (88.7%) of individual treatment, 10.9 of 12 h (90.8%) of group treatment, and 11.0 of 12 h (91.7%) of homework for a total of 48.5 of 54 treatment hours (89.8%). The average clinician-directed activities in SCORE were 36.1 (SD = 3.5) hours compared to 18.9 h in SMART. The average time a participant spent with a licensed clinician in SMART was half (51.8%) of that for the SCORE condition.

**Table 4:** Descriptive statistics detailing treatment hours per participant.

	SCORE^*^ (*n* = 43)	SMART (*n* = 51)	*p* val	Cohen’s *d*
Mean	48.5	18.9	<0.001	8.47
SD	4.6	1.8		
Median	43.0	20.0		
Range	36–54	11–20		

* Includes clinician directed and independent homework. Average hours with direct clinical care was 36.1 (SD = 3.5).

### Impact on cognition and GDS

Table 5 shows the results of a mixed-effect ANOVA examining change in GDS by treatment condition, which revealed a large within group effect insofar as all participants showed a significant change in GDS from T1 to T3 (*p* < 0.001, *η_p_*^2^ = 0.217). There was no significant interaction between condition and rate of change (*p* = 0.986, *η_p_*^2^ < 0.001). The greatest decline occurred between T1 (SCORE: *M* = 0.70, SD = 0.72; SMART: *M* = 0.70, SD = 0.79) and T2 (SCORE: *M* = 0.29, SD = 0.40; SMART: *M* = 0.29, SD = 0.58), with these treatment effects remaining at T3 (SCORE: *M* = 0.29, SD = 0.57; SMART: *M* = 0.28, SD = 0.52).

**Table 5:** Change in GDS by intervention-T1–T3.

	T1	T2	T3	Time		Time × Intervention	
	*M* (SD)	*M* (SD)	*M* (SD)	*p* val	*η_p_* ^2^	*p* val	*η_p_* ^2^
SCORE (*n* = 43)	0.70 (0.72)	0.29 (0.40)	0.29 (0.57)	<0.001	0.217	0.986	0.000
SMART (*n* = 51)	0.70 (0.79)	0.29 (0.58)	0.28 (0.52)				

*M, mean. SD, standard deviation. ηp2, partial eta squared.*

To maximize power, a secondary analysis was conducted that included any participant that completed T1 and T2 (Table 6). This increased the sample size for both SCORE (*n* = 49) and SMART (*n* = 65). The results of the repeated measures ANOVA were consistent with the primary analysis and again showed a large within group effect in terms of GDS improvement (*p* < 0.001, *η_p_*^2^ = 0.311) but no interaction (*p* = 0.400, *η_p_*^2^ = 0.008). The specific neuropsychological tests comprising GDS were also examined in this sample to describe the effects across domains. Table 6 shows that for the SMART intervention, there was a significant improvement in all cognitive measures with large effects (*d* > 0.50). For participants who completed SCORE there were significant improvements on all measures except the DKEFS Trail Making Condition 4, a measure of mental flexibility, but this approached significance (*p* = 0.058). The effect sizes associated with the SCORE intervention were large (*d* > 0.50) for 5 of 7 measures.

**Table 6:** Change in cognitive measures by intervention-T1–T2.

Measure	SCORE (*n* = 49)		SMART (*n* = 65)			
	T1	T2	T1	T2		
	Mean (SD)	Mean (SD)	Mean (SD)	Mean (SD)	*p* val	*d*
GDS	0.70 (0.72)	0.29 (0.40)			<0.001	0.652
SDMT^*^	−0.44 (0.89)	−0.05 (1.11)			0.014	−0.390
PASAT^*^	46.44 (10.13)	51.86 (10.79)			<0.001	−0.706
HVLT-R total recall^*^	39.12 (12.23)	46.51 (11.81)			<0.001	−0.671
HVLT-R delayed recall^*^	39.91 (14.42)	48.56 (10.77)			<0.001	−0.648
HVLT-R retention	43.23 (16.29)	51.40 (10.70)			0.005	−0.452
HVLT-R recognition index	43.62 (14.12)	47.02 (12.10)			0.156	−0.223
DKEFS TM condition 1	10.33 (2.93)	11.63 (2.38)			0.004	−0.471
DKEFS TM condition 2	10.77 (2.42)	12.16 (2.13)			<0.001	−0.663
DKEFS TM condition 3	10.37 (2.41)	12.42 (2.79)			<0.001	−0.763
DKEFS TM condition 4^*^	9.95 (2.20)	12.56 (9.09)			0.064	−0.290
DKEFS CW condition 1	7.74 (3.44)	9.70 (2.31)			<0.001	−0.866
DKEFS CW condition 2	9.26 (2.96)	10.00 (2.59)			0.024	−0.357
DKEFS CW condition 3^*^	9.09 (3.92)	10.84 (2.95)			<0.001	−0.549
DKEFS CW condition 4^*^	8.21 (3.26)	10.12 (2.85)			<0.001	−0.729
GDS			0.70 (0.79)	0.29 (0.58)	<0.001	0.597
SDMT^*^			−0.38 (1.04)	0.35 (1.26)	<0.001	−0.713
PASAT^*^			47.45 (10.25)	52.82 (10.52)	<0.001	−0.800
HVLT-R total recall^*^			40.59 (12.18)	46.41 (12.46)	0.002	−0.456
HVLT-R delayed recall^*^			39.61 (12.40)	48.04 (13.09)	<0.001	−0.639
HVLT-R retention			43.18 (12.83)	49.75 (10.10)	<0.001	−0.468
HVLT-R recognition index			40.73 (14.31)	46.98 (12.62)	0.003	−0.436
DKEFS TM condition 1			9.67 (3.15)	11.10 (2.08)	<0.001	−0.571
DKEFS TM condition 2			10.59 (2.89)	12.31 (2.12)	<0.001	−0.740
DKEFS TM condition 3			10.69 (2.57)	12.06 (2.25)	<0.001	−0.574
DKEFS TM condition 4^*^			9.78 (2.62)	10.82 (2.27)	<0.001	−0.564
DKEFS CW condition 1			7.49 (3.20)	9.16 (2.56)	<0.001	−0.605
DKEFS CW condition 2			9.39 (3.35)	10.41 (2.91)	0.008	−0.386
DKEFS CW condition 3^*^			8.14 (3.81)	10.61 (2.44)	<0.001	−0.792
DKEFS CW condition 4^*^			8.55 (3.43)	10.82 (2.47)	<0.001	−0.905

SDMT, Symbol Digit Modalities Test; PASAT, Paced Auditory Serial Addition Test; HVLT-R, Hopkins Verbal Learning Test Revised; DKEFS TM/CW, Delis-Kaplan Executive Function System Trail Making/Color Word interference. SD, standard deviation. *d*, Cohen’s *d*.

* Measures included in calculation of GDS.

### Impact on self-reported neurobehavioral symptoms

Table 7 shows the results of a mixed-effect ANOVA examining change in NSI total score by treatment condition revealed a significant within group effect where the overall sample had a reduction in NSI total score post-treatment (<0.001, *η_p_*^2^ = 0.138); however, the change was not impacted by the type of treatment (*p* = 0.412, *η_p_*^2^ = 0.010) (Table 7). The within group effect size was large with the change occurring from T1 to T2 and maintained at T3 (Table 7). Examination of the entire sample who completed T1 and T2 (Table 8) demonstrated that both interventions resulted in reduction of symptoms related to cognitive and vestibular functioning. The SCORE intervention but not the SMART intervention resulted in a reduction of affective symptoms. Neither intervention resulted in a reduction of somatosensory symptoms.

**Table 7:** Change in NSI by intervention, T1–T3.

	T1	T2	T3	Time		Time × Intervention	
	*M* (SD)	*M* (SD)	*M* (SD)	*p* val	*η_p_* ^2^	*p* val	*η_p_* ^2^
SCORE (*n* = 43)	39.53 (15.30)	33.81 (14.74)	33.72 (15.69)	<0.001	0.138	0.412	0.010
SMART (*n* = 48)	36.81 (12.29)	33.33 (12.74)	33.37 (13.48)				

*M, mean; SD, standard deviation; ηp2, partial eta squared.*

**Table 8:** Change in NSI summary and subscale scores by intervention-T1-T2.

Measure	SCORE (*n* = 43)		SMART (*n* = 50)			
	T1	T2	T1	T2	Within-group	
	Mean (SD)	Mean (SD)	Mean (SD)	Mean (SD)	*p* val	*d*
NSI total	39.53 (15.30)	33.81 (14.74)			<0.001	0.646
Affective	13.14 (5.57)	11.60 (5.16)			0.007	0.433
Cognitive	9.42 (3.46)	7.16 (3.55)			<0.001	0.729
Vestibular	3.67 (2.78)	3.05 (2.40)			0.016	0.384
Somatosensory	10.16 (5.07)	9.23 (4.94)			0.074	0.280
NSI total			37.18 (12.43)	34.00 (13.44)	0.027	0.327
Affective			12.78 (5.00)	11.74 (5.04)	0.061	0.271
Cognitive			8.86 (3.03)	7.70 (3.95)	0.010	0.379
Vestibular			3.35 (2.21)	2.84 (2.15)	0.023	0.337
Somatosensory			9.22 (4.55)	8.94 (3.83)	0.586	0.077

SD, standard deviation; *d*, Cohen’s *d*.

### Impact on self-reported functioning

Table 9 shows the results of a mixed-effect ANOVA examining change in KBCI total score by treatment condition revealed again a large within group effect (<0.001, *η_p_*^2^ = 0.377). Treatment improved patients’ perceived level of functioning from T1 to T2 and these gains remained stable at 3 months post-treatment. In addition, there was a significant interaction with a moderate effect size between change in KBCI total scores and treatment condition (*p* = 0.017, *η_p_*^2^ = 0.086). Further analysis revealed that while both interventions led to a reduction in KBCI total scores between T1 and T2, only individuals who received the SCORE treatment showed continued decrease in self-perceived functional difficulties from T2 To T3, while those individuals receiving SMART showed a modest regression. This change in either direction was approximately one standardized T Score point, and further analysis revealed that this difference was not statistically significant (*t*(92) = 1.97, *p* = 0.052, *d* = 0.407).

**Table 9:** Change in KBCI total scores by intervention, T1-T3.

	T1	T2	T3	Time		Time × Intervention	
	*M* (SD)	*M* (SD)	*M* (SD)	*p* val	*η_p_* ^2^	*p* val	*η_p_* ^2^
SCORE (*n* = 43)	70.17 (8.89)	66.18 (9.45)	65.28 (8.20)	<0.001	0.377	0.017	0.086
SMART (*n* = 50)	68.97 (6.60)	65.90 (8.07)	66.97 (8.35)				

*M, mean; SD, standard deviation; ηp2, partial eta squared.*

To maximize sample size, we examined all participants who completed T1 and T2 (Table 10). A medium sized effect was observed on the KBCI total for both interventions (0.652 vs. 0.710). For SMART all 8 scales that comprise the KBCI had a significant reduction with medium effect sizes. SCORE resulted in a significant reduction in 7 of 8 scales (the 8th scale, Impulsivity, approached significance).

**Table 10:** Change in summary and subscale KBCI scores by intervention-T1-T2.

Measure	SCORE (*n* = 49)		SMART (*n* = 64)			
	T1	T2	T1	T2	Within-group	
	Mean (SD)	Mean (SD)	Mean (SD)	Mean (SD)	*p* val	*d*
KBCI total	69.90 (8.72)	65.72 (9.29)			<0.001	0.652
Inattention	82.26 (10.10)	74.62 (11.95)			<0.001	0.830
Impulsivity	64.20 (13.17)	61.50 (10.64)			0.052	0.285
Interpersonal	62.67 (10.42)	59.56 (9.74)			<0.001	0.519
Apathy	67.99 (12.11)	64.60 (11.68)			0.004	0.432
Somatic	77.99 (12.50)	74.62 (11.50)			0.023	0.336
Unawareness	63.29 (10.63)	58.69 (11.34)			0.002	0.467
Communication	74.92 (13.67)	70.19 (12.84)			0.007	0.402
Emotional	65.87 (11.95)	62.02 (11.31)			<0.001	0.503
KBCI total			69.65 (7.58)	66.33 (8.73)	<0.001	0.710
Inattention			79.80 (9.85)	74.63 (10.92)	<0.001	0.658
Impulsivity			64.66 (10.57)	62.63 (10.94)	0.015	0.314
Interpersonal			63.77 (8.79)	61.73 (10.13)	0.016	0.311
Apathy			68.94 (10.87)	64.64 (11.87)	<0.001	0.710
Somatic			77.77 (12.34)	74.40 (11.82)	0.006	0.354
Unawareness			62.34 (9.41)	60.15 (10.05)	0.018	0.303
Communication			74.19 (11.35)	70.29 (12.68)	<0.001	0.452
Emotional			65.70 (10.79)	62.16 (11.94)	<0.001	0.494

SD, standard deviation; *d*, Cohen’s *d*.

### Relative cost effectiveness

Shown in Table 11 is the mean treatment time for each intervention as detailed above (SCORE: 42.4 h, SMART: 18.9 h). SLPs spent on average 10.6 h per participant in SCORE and 3.15 h per participant in SMART. Based on the most up-to-date labor statistics, we estimated that the hourly mean wage of a SLP was $52.39, while the hourly mean wage of the warfighter in this study was $34.06 (Table 12). We found that the mean cost of SCORE treatment was $1,999.47 compared to the mean cost of SMART treatment of $808.76, representing a cost savings of $1,190.71 per participant, or 60% reduction in cost (Table 12).

**Table 11:** Expected and mean observed time (hours) per treatment for SLPs and warfighters.

		Individual	Group	Homework	Transportation	Total
SCORE						
SLP	Expected time	30	3	–	–	33
	Actual time	26.6	2.73	–	–	29.33
Warfighter	Expected time	30	12	18	15	75
	Expected time	26.6	10.9	11	13	61.5
SMART						
SLP	Expected time	11	1.5	–	–	12.5
	Actual time	10	1.5	–	–	11.5
Warfighter	Expected time	11	9	–	8.5	28.5
	Expected time	10	9	–	8	27

SLP, speech language pathologist.

**Table 12:** Mean time cost per treatment.

	Mean wage	Mean time	Mean cost
SCORE			
SLP	$52.39	29.33	$1,536.60
Warfighter	$34.06	61.5	$2,094.69
			Total: $3,631.29
SMART			
SLP	$52.39	11.5	$602.49
Warfighter	$34.06	27	$919.62
			Total: $1,522.11

SLP, speech language pathologist.

## Discussion

This study directly compared two CR approaches for managing cognitive complaints in active-duty SMs with a history of mTBI and PCS-a novel metacognitive-focused treatment, “Strategic Memory Advanced Reasoning Training” (SMART), and a previously validated traditional program, the “Study of Cognitive Rehabilitation Effectiveness” (SCORE). The results of this prospective study showed that the two interventions had equivalent efficacy rehabilitating warfighters who had a history of mTBI with PCS. We showed that both interventions had a large effect on improving warfighter cognitive performance. The gains in cognitive performance, and an overall reduction in cognitive deficits, were observed immediately after the intervention and remained at 3 months post-intervention. In total, the results showed that completing cognitive rehabilitation reduced the rates of cognitive deficits in the sample by half. This improvement in cognitive performance was observed in terms of global functioning as well as on most individual cognitive tasks. This translates directly to warfighter cognitive readiness by helping diminish cognitive deficits that could impact the warfighter’s ability to complete their duties safely and effectively.

In addition to cognitive performance, the results also show that both CR programs were effective in reducing participants’ overall symptom burden as well as reducing their self-reported functional deficits. The improvements in self-reported functioning spanned all domains of interest including those directly related to cognitive abilities (i.e., inattention and impulsivity) as well as those indirectly related to military duties (i.e., interpersonal skills, communication with others, and emotional health). The one instance where we observed an interaction effect was on the KBCI, a measure of self-perceived functional difficulties. Notably, while both interventions reduced KBCI total scores between T1 and T2, those receiving the SCORE intervention showed continued improvement compared to participants in the SMART group, who showed a small regression toward baseline. However, when interpreting these results, we would caution that the change in either direction was only one T Score point, and the difference in change scores from T2 to T3 was not statistically significant suggesting this interaction is probably of limited meaningfulness.

Although the two treatments resulted in similar outcomes across multiple metrics, as anticipated, they varied dramatically in terms of treatment duration. This is important for two primary reasons. First, the quicker treatment can be completed, the less time the warfighter is away from their command, resulting in expedited return to duty. Second, shorter treatment time impacts the cost of healthcare. Cost effectiveness analysis showed SMART saved $1,190.71 per participant, representing a 60% reduction in cost. This streamlining of services has the potential to not only impact budget costs but is also likely to translate into better access to treatment for warfighters in need. This should be considered when identifying optimal treatments to improve and maintain warfighter brain health.

This study has some notable limitations. The design did not include a passive, no intervention control condition, which limits the generalizability of the findings. Inclusion of an additional active control (e.g., computerized CR program) would have also strengthened the conclusion drawn from this study. However, given the extant literature on cognitive rehabilitation, the improvements observed are believed to be directly related to the intervention and not external non-treatment factors. Particularly, the improvements on the cognitive tasks are generally larger than what is observed from practice effects. The fact that cognitive improvements were observed in conjunction with self-reported changes on two independent measures also supports the veracity of the results. Lastly, the 60-h SCORE protocol used in this study was derived from (13), which was current at the time of this study’s inception. However, since that time, it is likely that efforts have been made to revise and streamline SCORE, as well as other CR programs. Future research should focus on evaluating the effectiveness of shorter or more intensive versions of CR protocols to determine whether they can produce comparable cognitive and behavioral outcomes in SMs with mTBI and PCS.

The military has a longstanding desire to raise mental agility and acumen in its military operators, with a key goal for warfighters to return to duty. Persistent symptoms of concussion may delay a SM’s return to full active-duty status. Importantly, it is worth mentioning that mild or absence of impairment on neuropsychological assessments does not always correlate with patients’ symptoms, particularly in cases of mTBI/concussion. Consequently, detection and treatment of such subtle impairments associated with mTBI continues to be a challenge for clinicians. Despite some successful CR interventions many SMs continue to present with deficiencies in daily functioning (whether treated or not) and psychological wellbeing secondary to the effects of mTBI. Given SMART’s effectiveness (as demonstrated by reduced cognitive deficits, improved performance on cognitive tasks) and reduced time of treatment compared to traditional CR in this study, SMART is a cost effective and feasible alternative to traditional CR programs for treating PCS in active duty SMs with the goal of improving overall cognitive fitness/readiness for warfighters.

## Data Availability

The raw data supporting the conclusions of this article will be made available by the authors without undue reservation.
